# Probing Quantum Confinement and Electronic Structure at Polar Oxide Interfaces

**DOI:** 10.1002/advs.201800242

**Published:** 2018-06-24

**Authors:** Danfeng Li, Sébastien Lemal, Stefano Gariglio, Zhenping Wu, Alexandre Fête, Margherita Boselli, Philippe Ghosez, Jean‐Marc Triscone

**Affiliations:** ^1^ Department of Quantum Matter Physics University of Geneva 24 quai Ernest‐Ansermet CH‐1211 Geneva 4 Switzerland; ^2^ Theoretical Materials Physics Q‐MAT CESAM Université de Liège B‐4000 Liège Belgium; ^3^ State Key Laboratory of Information Photonics and Optical Communications and School of Science Beijing University of Posts and Telecommunications Beijing 100876 China

**Keywords:** 2D superconductivity, electronic structure, oxide interfaces, polar discontinuity, quantum confinement

## Abstract

Polar discontinuities occurring at interfaces between two materials constitute both a challenge and an opportunity in the study and application of a variety of devices. In order to cure the large electric field occurring in such structures, a reconfiguration of the charge landscape sets in at the interface via chemical modifications, adsorbates, or charge transfer. In the latter case, one may expect a local electronic doping of one material: one example is the two‐dimensional electron liquid (2DEL) appearing in SrTiO_3_ once covered by a polar LaAlO_3_ layer. Here, it is shown that tuning the formal polarization of a (La,Al)_1−_ *_x_*(Sr,Ti)*_x_*O_3_ (LASTO:*x*) overlayer modifies the quantum confinement of the 2DEL in SrTiO_3_ and its electronic band structure. The analysis of the behavior in magnetic field of superconducting field‐effect devices reveals, in agreement with ab initio calculations and self‐consistent Poisson–Schrödinger modeling, that quantum confinement and energy splitting between electronic bands of different symmetries strongly depend on the interface total charge densities. These results strongly support the polar discontinuity mechanisms with a full charge transfer to explain the origin of the 2DEL at the celebrated LaAlO_3_/SrTiO_3_ interface and demonstrate an effective tool for tailoring the electronic structure at oxide interfaces.

Functionalities offered by the interfaces between different materials have been the fundamental basis of modern electronic devices and information technology.^1^, ^2^ Transition metal oxides (TMOs), largely owing to their correlated d electrons and entanglement of various degrees of freedom, provide an ideal platform for establishing a variety of novel electronic properties.^3^, ^4^ In recent years, a burst of research activities on heterostructures of transition metal oxides has been driven by the quest for emergent phenomena at interfaces that are absent in the bulk parent compounds.^5^ Indeed, at the interface, different phenomena are at play, including symmetry breaking, electrostatic coupling, atomic rearrangement, etc.^6^, ^7^ One phenomenon particularly relevant for transition metal ions with multiple valence states is charge transfer. This effect has been suggested to be the origin of several recently discovered novel electronic states: its driving force has been attributed to differences in cationic electron affinity, for instance, in LaNiO_3_/LaMnO_3_ 8, 9 or CaMnO_3_/CaRuO_3_,^10^, ^11^ or to charge delocalization in LaTiO_3_/SrTiO_3_ 12, 13 or LaMnO_3_/SrMnO_3_ 14, 15 heterostructures.

For the LaAlO_3_ (LAO)/SrTiO_3_ (STO) interface, the polar discontinuity has often been regarded as the origin of the charge transfer^16^, ^17^, ^18^, ^19^ and the formation of a superconducting 2D electron liquid (2DEL).^20^, ^21^ The formal polarization of LAO due to the (LaO)^+^ and (AlO_2_)^−^ atomic planes is disrupted along the [001] direction by the nonpolar STO, since (SrO)^0^ and (TiO_2_)^0^ planes are formally charge neutral. Density‐functional theory (DFT) calculations^22^, ^23^ for a defect‐free interface show that the buildup of the electric potential inside LAO leads to a Zener breakdown^17^ and a progressive charge transfer of 0.5 electrons per surface unit cell (0.5 e^−^ per u.c.) from the LAO surface O‐2p states to the interfacial Ti‐3d states, thus forming a 2DEL. This mechanism successfully explains the observed threshold LAO thickness, *t*_c_, of 4 u.c. for the onset of conductivity,^18^ but faces questions raised by several experiments, reporting, for instance, the lack of O‐2p hole pockets^24^ or a weak initial electric field in LAO.^25^, ^26^, ^27^ Relying on polar discontinuity and the formation of oxygen vacancies at the LAO surface, recent theoretical work^28^, ^29^, ^30^, ^31^ predicts a similar critical thickness *t*_c_, while solving some of the discrepancies mentioned above.

Reinle‐Schmitt et al.^32^ showed that polar discontinuity and critical thickness are indeed correlated: tuning the polar discontinuity by alloying LAO with STO [(La,Al)_1−_ *_x_*(Sr,Ti)*_x_*O_3_, denoted as LASTO:*x*], *t*_c_ increases, with an inverse proportionality to the fraction of LAO in the alloy and therefore with the formal polarization, in perfect agreement with DFT predictions. The self‐confinement of the transferred charge leads to an electronic reconstruction of the Ti t_2g_ bands, increasing the energy of d*_xz_*_/_ *_yz_*‐symmetry states by ≈50 meV with respect to the d*_xy_* states.^33^, ^34^ This band structure differs from the electronic structure of bulk STO,^35^ and is at the origin of the unique electronic properties of the LAO/STO interface. However, the carrier density (*n*_2D_) measured using Hall effect at the LASTO:0.5/STO interface was found to be comparable to the one of the standard LAO/STO interface (of the order of ≈10^13^ cm^−2^),^32^ a value significantly lower than the theoretical prediction based on the polar discontinuity scenario (*n*_2D_ = 1.7 × 10^14^ cm^−2^ or 0.25 e^−^ per u.c. for the LASTO:0.5/STO interface and 3.3 × 10^14^ cm^−2^ or 0.5 e^−^ per u.c. for the LAO/STO interface). This discrepancy between the predicted and measured carrier densities has been one important open issue questioning the origin of the 2DEL and has been related to different mechanisms, such as charge localization^22^, ^36^, ^37^ due to interface disorder, surface defects, and selective quantum confinement in a single layer, or to antisite defects,^30^ or to phase separation.^38^

Here, we probe the quantum confinement and its consequences on the electronic band structure at the superconducting LASTO:0.5/STO interface and compare the results with standard LAO/STO interfaces. Using sophisticated superconductivity measurements and DFT‐complemented Poisson–Schrödinger modeling, we provide strong evidences that, although transport measures a fraction of the carriers predicted by the polar discontinuity scenario, the full amount of charge is indeed present at the interfaces. The field‐effect tuning of the superconducting state suggests that the energy splitting between bands of different symmetries (d*_xy_* vs d*_xz_*_/_ *_yz_*) is reduced with the widening of the confining potential, in agreement with the models. Our results strongly support that charges with nominal carrier densities of 0.5 and 0.25 e^−^ per u.c. for the LAO/STO and LASTO:0.5/STO interfaces, respectively, contribute to the electronic confinement and only these total carrier densities can explain the differences in the extension of the 2DEL and in their electronic configurations.

**Figure** **1**a shows a schematic of the atomic structure of the LASTO:0.5/STO interface with the La/Sr and Al/Ti cations spread evenly on the AO and BO_2_ perovskite planes according to energy calculations performed by DFT.^32^ Consequently, along the [001] direction, the layer has alternating planes with formal electronic charges of +0.5 and −0.5 per u.c. The polar discontinuity is therefore half the one of standard LAO/STO interfaces. As a result, the density of the transferred charges to the interface is estimated to be 0.25 e^−^ per u.c. (≈1.7 × 10^14^ cm^−2^). LASTO:0.5 films were grown by pulsed‐laser deposition (PLD) using growth conditions described in the Experimental Section. The oscillations of the reflection high‐energy electron diffraction (RHEED) intensity provide a measure of the thickness of the layer during the growth, as shown in Figure 1c for a 10 u.c. film. Topographic scan using atomic force microscopy (AFM) shows a sharp step‐and‐terrace structure on the film surface with single‐unit‐cell step height (Figure 1c inset). Both RHEED and AFM data indicate a high growth quality of the films. We note that, in order to probe the intermixing strength and the sharpness of the LASTO:0.5/STO interface, detailed scanning transmission electron microscopy (STEM) study is required. Hall bars (see Figure 1b) were defined using a prepatterning technique for transport measurements.^39^ A gate electrode was deposited at the backside of the STO substrate (see Figure 1b) in order to tune the properties of the electron liquid. Transport measurements were performed in a dilution cryostat equipped with a rotating sample holder, allowing the orientation of the magnetic field to be varied from perpendicular to parallel to the interface plane.

**Figure 1:**
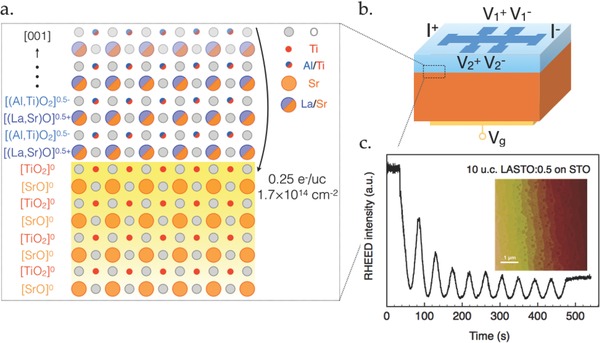
Schematics of the LASTO:0.5/STO structure and sample preparation. a) A detailed illustration of the interfacial structure with atomic arrangements and charges per atomic plane. Electrons with surface density of 0.25 e^−^ per u.c. are expected to be transferred. b) A sketch of the LASTO:0.5/STO field‐effect device (5 × 5 mm^2^) with the back‐gate electrode used to apply the gate bias (*V*_g_) facing the 500 µm wide Hall bar. c) Oscillations of in situ RHEED intensity during the growth of a 10 u.c. thick LASTO:0.5 layer and (inset) its surface topography acquired by atomic force microscopy (AFM), revealing the step‐and‐terrace morphology.

A superconducting ground state is observed when the sample is cooled below a critical temperature, *T*_c_, of ≈200 mK, generally lower than that of the LAO/STO system (see later in the discussion).^21^ In **Figure** **2**b, we show the critical magnetic field *H^∗^*(*T*) for perpendicular and parallel orientation of a LASTO:0.5/STO interface in its virgin state. The critical temperature for each magnetic field was defined as the temperature at which the sheet resistance *R*_s_ reaches half the normal state value (estimated at 500 mK). We calculate the in‐plane coherence length ξ_∥_(*T*) from the Ginzburg–Landau *H*_c2_ formula using the perpendicular field (Equation (1)) and we extrapolate for *T* = 0 a value of ≈55 nm, similar to the one observed for standard interfaces.^40^

(1) $$\xi_{∥}\left(T\right)\;\;=\;\;\sqrt{\frac{\Phi_{0}}{2\pi \mu_{0}H_{⊥}^{*}\left(T\right)}}$$

**Figure 2:**
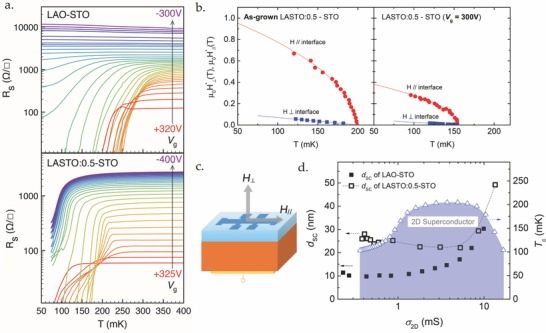
Superconducting properties and phase diagram of the LASTO:0.5/STO interface. a) Field‐effect modulation of sheet resistance (*R*_s_) as a function of *T* on a semilogarithmic scale of the LASTO:0.5/STO interface (bottom panel), in comparison with the LAO/STO interface (top panel, replotted from ref. 21). For LASTO:0.5/STO, the gate voltage varies from −400 to +325 V in steps of 25 V. b) Perpendicular (dots) and parallel (squares) critical fields (*H*_⊥_ *^∗^*, *H*_∥_ *^∗^*) as a function of temperature (*T*) for the as‐grown state (left panel) and for a state with *V*_g_ = 300 V (right panel) for LASTO:0.5/STO. The solid line for H_∥_ *^∗^* is a fit to the data using the Ginzburg–Landau formula for a 2D film (see text), while the line for H_⊥_ *^∗^* is a guide to the eye. c) Configurations for the measurements of superconducting critical fields applied perpendicular and parallel to the interface plane. d) Phase diagram of the LASTO:0.5/STO interface, showing in blue the superconducting state, plotted as a function of the normal‐state sheet conductance. The superconducting layer thickness *d*_SC_ (left axis, black open square) is compared to that of the LAO/STO interface^59^ (left axis, black solid square). The black dotted line is a guide to the eye.

The temperature dependence of the parallel critical field *H*_∥_ *^∗^*(*T*) follows the 2D behavior of a superconducting thin film *H*_∥_ *^∗^*(*T*) ∝ [1 − *T*/*T*_c_(*H* = 0)]^1/2^.^41^ We then use Equation (2) for a 2D superconductor to calculate its thickness (*d*_SC_) from the value of *H*_∥_ *^∗^*(*T*).

(2) $$d_{\mathrm{SC}}\;\;=\;\;\frac{\sqrt{3\;}\Phi_{0}}{\pi \xi_{∥}\left(T\right)\mu_{0}H_{∥}^{*}\left(T\right)}$$

We obtain *d*_SC_ ≈ 24 nm, independent of the temperature as expected, a value that is larger by a factor of 2 than the one of LAO/STO interfaces.^40^, ^42^, ^43^ We note that the thickness obtained from the analysis of the anisotropic superconducting properties is a characteristic thickness which, in the case of pure LAO/STO, was found to agree well with the vertical spread of the electron system determined using other methods.^42^, ^43^

Field effect was then used to tune the doping level and the superconducting properties of the interface. Figure 2a shows the modulation of the superconducting transitions when the gate voltage (*V*_g_) is varied from −400 to +325 V, in comparison with the data of the LAO/STO interface.^21^ We observe that *T*_c_ and the normal state resistance (see the Supporting Information for the *R*_s_ vs *V*_g_ plot) are effectively tuned; we note however that for the largest negative voltages, i.e., in the strongest depletion regime, the system remains metallic and superconducting and we do not attain the insulating state reported for standard LAO/STO interfaces (top panel in Figure 2a). The presence of superconductivity and relatively high conductance at the largest negative voltages can be related to the occupation of d*_xz_*_/_ *_yz_*‐symmetry states, even at the lowest doping levels, at the LASTO:0.5/STO interface and will be discussed below. The evolution of *T*_c_ versus σ_2D_ (sheet conductance in the normal state), illustrated in Figure 2d, shows a dome‐like behavior, reaching a maximum of 206 mK. For several gate voltages, the parallel and perpendicular critical magnetic fields were measured: data for *V*_g_ = 300 V is shown in the right panel of Figure 2b. These measurements allow us to estimate the superconducting layer thickness across the phase diagram. This information is shown in Figure 2d, where the superconducting thickness for LASTO:0.5/STO samples is displayed in comparison with LAO/STO interfaces. We see that the thickness for the alloy samples is larger than that of standard interfaces across the phase diagram: *d*_SC_ of LASTO:0.5/STO interfaces falls in the range of 20–30 nm for a wide interval of conductance, while it remains 10–15 nm for the LAO/STO interface. Assuming that this superconducting thickness mirrors an effective width of the confining potential, we attribute this enhancement to the difference in the transferred charge due to a modified polar discontinuity.

To support this idea, we model the quantum confinement at these oxide interfaces using two complementary approaches, first‐principle calculations and Poisson–Schrödinger (P–S) approach.^44^

Previous DFT studies on LAO/STO interfaces^34^, ^44^, ^45^, ^46^, ^47^, ^48^, ^49^ have investigated the fully compensated interface (3.3 × 10^14^ cm^−2^) through symmetric superlattices (with two n‐type interfaces). Lower carrier densities have been reached by artificially removing some charges at the interface and compensating by a positive background.^34^, ^48^ In our study, the interfacial charge is directly modified by explicitly tuning the polar discontinuity, i.e., by switching LAO to LASTO:0.5 solid solution. In the following text, we present the two doping cases, 0.5 and 0.25 e^−^ per u.c. for (LAO)_2_/(STO)_30_ and (LASTO:0.5)_2_/(STO)_30_ superlattices (each with 330 atoms, see the Supporting Information). For the purpose of describing the charge profile at the interface, we set the extension of the STO block as wide as possible for numerical calculations (much larger than previous studies^34^, ^48^). The calculations also provide physical parameters used later in the P–S modeling.

**Figure** **3** illustrates a sketch of the LASTO:0.5/STO system (Figure 3a) and compares the main results from the calculations. The dashed line indicates the position of the interface. The DFT computed charge profile *n*_3D_(*z*) for the two interfaces is displayed in Figure 3b. The vertical dashed dotted line denotes the extension of the largest STO supercell. Performing calculations for different sizes of the STO supercell, we have noticed that the charge profile extends further into the substrate each time we increase the supercell width, but this occurs on a logarithmic charge density scale (see the Supporting Information). Looking at the plot, we see that most of the carriers are located in the first unit cells in both cases; moving away from the interface, a crossing between the two profiles occurs (at *z* ≈ 3.5 nm): for lower carrier density, the charge is more spread into the supercell, while for higher density, it is more localized close to the interface. These calculations rely on the self‐consistent DFT dielectric constant (ε_STO_ = 250 in zero field) that reflects the behavior of STO at room temperature.

**Figure 3:**
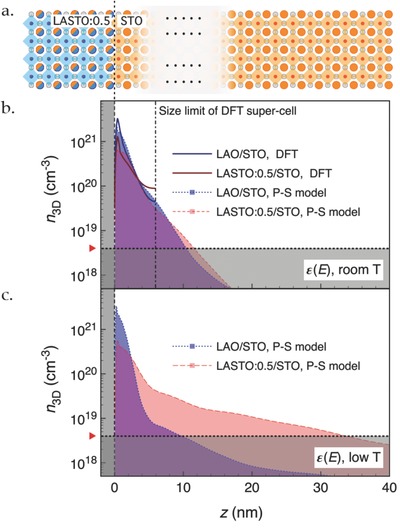
Charge distributions in STO calculated for the two interfaces. The panels display the charge density (*n*_3D_) in logarithmic scale as a function of distance (*z*) from the interface for *n*_2D_ = 0.5 e^−^ per u.c. (LAO/STO) and 0.25 e^−^ per u.c. (LASTO:0.5/STO). a) Schematic of atomic structures of the LASTO:0.5/STO interface. b) Results from DFT calculations (solid lines) and P–S model (dotted/dashed lines) for a room‐temperature field‐dependent STO dielectric constant ε(*E*) (see ref. 71 and the Supporting Information). c) Charge profile estimated with the P–S model (dotted/dashed lines) using the low‐temperature ε(*E*) (Supporting Information). Black dashed line indicates the interface. Dashed dotted line corresponds to the size limit of the largest supercell used in DFT calculations. Dotted lines and the red triangles indicate the 3D density threshold (*n*_3D,th_ ≈ 4 × 10^18^ cm^−3^) for the occurrence of superconductivity in STO.

To access the low temperature case which is experimentally measured, we need to overcome some limitations of the DFT approach: i) the STO size limit in the numerical calculations and ii) the low temperature diverging value of the dielectric constant ε_STO_ that is not captured. To bypass these limitations, a self‐consistent P–S model using DFT data is employed.^50^ Effective mass *m^∗^* values are obtained from DFT calculations. We notice that the effect of the renormalization of *m^∗^* due to, for instance, polaronic effects,^43^, ^51^ has also been examined (see the Supporting Information): while this renormalization indeed slightly affects the calculated charge distribution, it does however not alter the main findings, discussions, and conclusions below. The field dependence of the dielectric constant ε_STO_(*E*) is adjusted according to the measurements^52^ (details can be found in the Experimental Section and the Supporting Information).

The resulting profile for the room‐temperature configuration (ε_STO_ = 250) is first compared with the DFT data in Figure 3b: the two approaches provide similar charge density profiles (especially in the regime close to the interface), therefore validating the P–S model with respect to the DFT calculations; it further highlights that, although large and at the computation limit, the size of the DFT supercells is still too small to correctly describe the tail of the carrier density profile, which is better accessible in the P–S simulation. From the latter, we determine that for both surface carrier densities, the spatial extension at the 3D density threshold for the occurrence of superconductivity (*n*_3D,th_ ≈ 4 × 10^18^ cm^−3^, labeled in Figure 3b)^53^ is ≈10 nm. We note that the thickness of the 2DEL estimated by different techniques differs a lot – from a few u.c. to ≈10 nm.^42^, ^54^, ^55^, ^56^ This may be due to the high density in the first few atomic layers of STO that is decaying by a factor of 10 in 5 nm. The tail of this distribution may fall below the sensitivity of some of the techniques used to probe the 2DEL thickness.

At low temperature, ε_STO_ increases due to the quantum paraelectric behavior of STO: in this regime, the field dependence is described by Equation (3) (see the Experimental Section and Figure S3 in the Supporting Information for details). Using this dependence, the charge density profiles *n*_3D_(*z*) are calculated using the P–S model for the two interfaces and are illustrated in Figure 3c. We see that the effect of the large ε_STO_ is to open the confining potential for the LASTO:0.5/STO interface with more charges spreading into STO. At the *n*_3D,th_ (labeled in Figure 3c), the extension of the 2DEL is ≈10 nm for the LAO/STO interface and 31 nm for the LASTO:0.5/STO interface, values that agree with the estimation of the superconducting thickness extracted from the critical field measurements discussed above. This suggests that the change in superconducting thickness and the confinement scale at the LASTO:0.5/STO interface is due to a reduced total charge density originating from a modified polar discontinuity (see discussion below). The modeling also clearly shows that the dielectric properties of STO and the total 2D carrier density define the confinement of the charge, in agreement with a recent tight‐binding study.^57^ We note that, at low temperature, the P–S modeling gives a lower integrated charge density for *n*_3D_(*z*) above *n*_3D,th_ (blue area above the dotted line in Figure 3c as compared to that in Figure 3b). This is also due to the remarkable temperature dependence of ε_STO_(*T*) and the large electric field dependence of ε_STO_(*E*) at low temperature: a portion of the charges extends further into STO, with density below *n*_3D,th_.

The effect of the surface carrier density and the quantum confinement on the electronic band structures calculated from DFT for the two superlattices (STO)_30_/(LAO)_2_ and (STO)_30_/(LASTO:0.5)_2_ is displayed in **Figure** **4**a,b. The Fermi level lies at 0 eV and the symmetry of the electronic states (d*_xy_* and d*_xz_*_/_ *_yz_*) is indicated by an arrow. We see changes in the splittings between sub‐bands as well as between bands with different symmetry: for a larger extension of the 2DEL (LASTO:0.5/STO, panel (b)), the sub‐band spacing is reduced and the d*_xz_*/d*_yz_* bands are occupied at lower densities. The calculated bottom of the first d*_xz_*_/_ *_yz_* band relative to Fermi level depends on the size of the superlattices, which is discussed in Figure S8 (Supporting Information).

**Figure 4:**
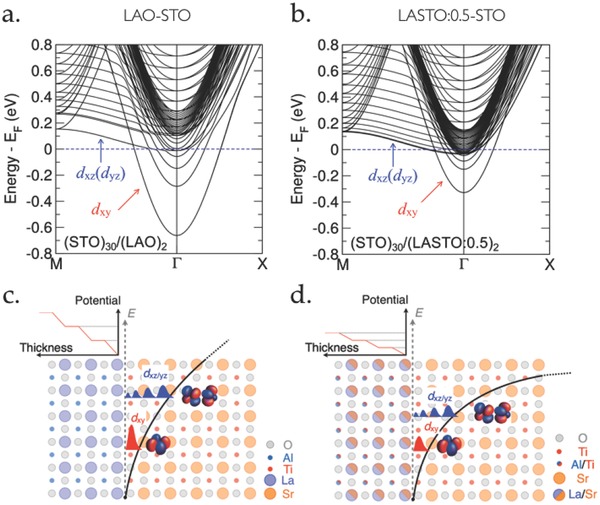
Electronic structure of the two interfaces. Band structures of a) the LAO/STO and b) the LASTO:0.5/STO interfaces, calculated by DFT. The t_2g_ bands are labeled according to their symmetry (d*_xy_*, d*_xz_*_/_ *_yz_*). Schematics of the atomic arrangements, built‐in electric potential (red lines), quantum confinement potential (black lines), and d*_xy_*–d*_xz_*_/_ *_yz_* band splittings of c) the LAO/STO and d) the LASTO:0.5/STO interfaces. For a larger extension of the 2DEL, the band splitting is reduced.

The agreement between the experimental estimation of the superconducting layer thickness and the theoretical calculations provides strong support to the polar discontinuity as the doping mechanism of the interfaces. Despite the similar mobile carrier densities extracted from Hall measurements for the two interfaces,^32^ calculations and superconducting critical fields measurements show that the increase in the characteristic thickness of the 2DEL for the LASTO:0.5/STO interface is a direct result of the reduced total transferred charges (from 0.5 to 0.25 e^−^ per u.c.) upon modification of the polar discontinuity. Although a large fraction of these charges does not contribute to electric transport, it defines the strength of the electric field in STO and consequently its effective dielectric constant at the interface, that finally sets the extension of the 2DEL. In fact, considering only a charge density extracted from Hall measurements (≈0.05 e^−^ per u.c.) at the interfaces in P–S calculations would result in an ineffective electric field and deconfinement of the electrons with the spatial extension over ≈60 nm at density of *n*_3D,th_. This demonstrates that all charges with nominal charge density predicted by the polar discontinuity mechanism are transferred to the interface. Our results show significant differences from the work by Ueno et al.,^58^ where, experimentally, no dependence of the superconducting thickness on the electrostatically tuned carrier density was observed.

The consequence of the larger charge profile at the LASTO:0.5/STO interface is a band structure with larger contribution of the d*_xz_*_/_ *_yz_* bands to the density of states, as shown in Figure 4b. We argue that this electronic configuration is the reason for the modified phase diagram of the superconducting state of the LASTO:0.5/STO interface presented in Figure 2. The overall lower *T*_c_ observed in the system, as compared with that of the LAO/STO interface, can be due to a shallow confining potential, which gives a reduced effective *n*_3D_ as the extension increases.^59^, ^60^ Furthermore, we observe that field effect is not efficient enough to deplete the 2DEL in order to suppress superconductivity nor to reach a highly resistive state. This is clearly different from the LAO/STO system. The presence of superconductivity and the low resistance state in the depletion regime suggest that the d*_xz_*_/_ *_yz_*‐symmetry states are occupied even at low dopings due to reduced energy splittings between bands, highlighting the relevance of these orbitals on superconductivity and high electron mobility at the interfaces.

Our study provides a direct comparison between ab initio calculations performed on a massive supercell and the self‐consistent P–S modeling, revealing that the latter method is also valid to describe quantum confinement of oxide systems where 3d electronic states are more spatially localized than p bands in semiconductor. The ab initio calculations of the effective masses combined with the experimental determination of the dielectric properties allow the P–S modeling to describe the field and charge configuration on scales that today cannot be achieved by DFT methods.

To conclude, we have demonstrated that by doping LAO films with STO to form a 50% alloy compound, we are able to successfully change the polar discontinuity at this (super)conducting interface. This modulation leads to a significant change in the interfacial confining potential that we have estimated from the measurements of the characteristic superconducting thickness. The evolution of the confinement with the change in polarization is captured by advanced large‐scale DFT calculations and self‐consistent Poisson–Schrödinger modeling, only when assuming that a full transfer of charges takes place at the LAO/STO interface with the density predicted by the polar catastrophe model using first‐principle calculations. The resulting band structure for the LASTO:0.5/STO interface reveals a larger contribution of the d*_xz_*_/_ *_yz_* bands to the density of states and explains the persistence of the superconducting state in the depletion regime. This study shows that the control of the polar discontinuity at oxide interfaces by chemical composition is an effective tool for engineering novel electronic states in these compounds.

## Experimental Section

*Sample Preparation*: Prior to film deposition, Hall‐bar patterns with the crystalline TiO_2_‐terminated surface of (001) single‐crystal STO substrates were defined using photolithography, followed by a subsequent amorphous STO deposition used as hard mask and lift‐off process. Films were then grown by pulsed‐laser deposition using standard growth conditions:^61^ a KrF laser (248 nm) with a pulse energy of 40 mJ (≈0.8 J cm^−2^), repetition rate of 1 Hz; growth temperature of 800 °C, O_2_ pressure of 1 × 10^−4^ mbar; samples were cooled after growth to 540 °C in 200 mbar O_2_ and maintained at this temperature and pressure for 1 h before being cooled down to room temperature in the same atmosphere. The growth rate was ≈55 laser pulses per monolayer. The deposition was fully monitored by RHEED and specular spot intensity oscillations indicated a layer‐by‐layer growth. Rutherford backscattering spectrometry (RBS) analysis revealed a film stoichiometry in agreement, within the experimental uncertainties (1.5%), with the nominal concentration of *x* = 0.5 for a 40 nm thick film.

*Field‐Effect Device and Superconductivity Measurements*: Aluminum wires were ultrasonically bonded to the sample. Gold pad was deposited by sputtering on the backside of the sample as back‐gate electrode. A dc bias was applied across the STO substrate between back gate and the conducting interface. Superconductivity measurements were performed in a ^3^He/^4^He dilution refrigerator (Leiden Cryogenics) with a base temperature of 50 mK and a superconducting magnet allowing field of up to 15 T to be reached. Samples were attached to a rotator head for anisotropic magnetic field measurements. Precise parallel and perpendicular orientations with respect to the magnetic field were determined using both longitudinal resistance *R_xx_* and Hall resistance *R_xy_*. In the parallel direction, an off‐axis angle is estimated to be smaller than 0.02° from the *R_xy_* signal. Current was applied to the conducting channel from a Keithley 6220 high precision current source. Voltages were recorded using Keithley 2182 nanovolt meters.

*DFT Calculations*: First‐principle calculations were performed with the CRYSTAL code,^62^ which implements the Kohn–Sham ansatz to density functional theory^63^ using a linear combination of atomic orbital (LCAO) approach and local Gaussian basis sets. Electronic exchange‐correlation effects were described with the B1‐WC hybrid functional,^64^ including 16% of Hartree–Fock exact exchange. Sampling of the Brillouin zone with a Monkhorst–Pack^65^ 6 × 6 × 1 *k*‐point mesh ensured a proper convergence of the total energy at the self‐consistent field level, with a threshold criterion of 10^−8^ Ha. The electronic properties were computed using a refined 12 × 12 × 2 *k*‐point mesh. A Gaussian smearing of the Fermi surface was set to 0.001 Ha. The basis sets used for different atoms are detailed in ref. 66 for Ti,^67^ for O,^68^ for Al, and^69^ for La, and correspond to the ones used in ref. 32. The optimization of the atomic positions was performed with convergence criteria of 1.5 × 10^−4^ Ha Bohr^−1^ in the root‐mean square values of the energy gradients, and 1.2 × 10^−3^ Bohr in the root‐mean square values of the atomic displacements. The evaluation of the Coulomb and exchange series was determined by five parameters, fixed to their default^70^ values: 7, 7, 7, 7, and 14. Calculations were performed on off‐stoichiometric (STO)_30_/(LAO)_2_ and (STO)_30_/(LASTO:0.5)_2_ superlattices, with two n‐type interfaces, with an additional TiO_2_ plane in STO, and an additional LaO (LaSrO) plane in LAO (LASTO). The effective masses associated to the different Ti t_2g_ bands were calculated. Each t_2g_ band had two light effective masses associated to the light carriers and one heavy effective mass associated to the heavy carriers (for Ti d*_xy_*, *m^∗^_xx_* = *m^∗^_yy_* = *m^∗^*_L_ and *m^∗^_zz_* = *m^∗^*_H_). With both superlattices, we have *m^∗^*_L_ = 0.4 *m*_e_ and *m^∗^*_H_ = 5.9 *m*_e_ (very close to the calculated values in cubic STO: *m^∗^*_L_ = 0.4 *m*_e_ and *m^∗^*_H_ = 6.1 *m*_e_).

*P–S Modeling*: Starting from the band structure at the LAO/STO interface suggested by DFT calculations,^34^ the confinement, energy levels, and charge distribution at both interfaces were modeled by self‐consistently solving the Poisson and Schrödinger equations.^50^ For the boundary conditions of the potential profile *V*(*z*), it was considered that the LAO or the LASTO:0.5 layer fixes the displacement field *D* at the interface to be *D* = *n*_2D_ *e* with *n*_2D_ = 0.5 or 0.25 e^−^ per u.c., respectively, while *D* = 0 at the bottom side of STO. The static dielectric constant of STO calculated by DFT ($\varepsilon_{\mathrm{STO}}^{\mathrm{DFT}}$(*E* = 0) = 250) was used as the room‐temperature static STO ε_STO_(*E* = 0). The low‐temperature static ε_STO_(*E* = 0) was obtained from a fit to our experimental data (ε_STO_(*E* = 0) = 25 462). For both the temperatures, the high‐field dependent ε_STO_(*E*) was estimated from the work of Stengel.^71^ ε_STO_(*E*) takes the general form:

(3) $$\varepsilon_{\mathrm{STO}}(E)\;\;=\;\;1\;\;+\;\;\frac{B}{{\left[1+{\left(E/E_{0}\right)}^{2}\right]}^{1/3}}$$

with *B* = 250, *E*_0_ = 10^7^ V m^−1^ for room temperature, and *B* = 25 462, *E*_0_ = 82213 V m^−1^ for low temperature, respectively (see the Supporting Information).

For details of the calculation, the algorithm used for convergence of the self‐consistency calculations, refer to ref. 72. The field dependence of ε_STO_(*E*) can be found in the Supporting Information.

## Conflict of Interest

The authors declare no conflict of interest.

## Supporting information

Supplementary
