# Supplementary material for: Probing Quantum Confinement and Electronic Structure at Polar Oxide Interfaces
Source: Adv Sci (Weinh). 2018 Jun 24;5(8):1800242. doi: 10.1002/advs.201800242 (PMC6097152; doi:10.1002/advs.201800242)
Supplement: Supplementary file 1 — Supplementary [file ADVS-5-1800242-s001.pdf]

Copyright WILEY-VCH Verlag GmbH & Co. KGaA, 69469 Weinheim, Germany, 2018.

Supporting Information

for *Adv. Sci.,* DOI: 10.1002/advs.201800242

Probing Quantum Confinement and Electronic Structure at Polar Oxide Interfaces

Danfeng Li, Sébastien Lemal, Stefano Gariglio, Zhenping Wu, Alexandre Fête, Margherita Boselli, Philippe Ghosez, and Jean-Marc Triscone*

Copyright WILEY-VCH Verlag GmbH & Co. KGaA, 69469 Weinheim, Germany, 2016.

Supporting Information

Probing Quantum Confinement and Electronic Structure at Polar Oxide Interfaces

Danfeng Li†, Sébastien Lemal, Stefano Gariglio, Zhenping Wu, Alexandre Fête, Margherita Boselli, Philippe Ghosez, and Jean-Marc Triscone*

Dr. Danfeng Li, Dr. Stefano Gariglio, Dr. Zhenping Wu, Dr. Alexandre Fête, Margherita Boselli, Prof. Jean-Marc Triscone
Department of Quantum Matter Physics, University of Geneva, 24 quai Ernest-Ansermet, CH-1211 Geneva 4, Switzerland
E-mail: denverli@stanford.edu

Sébastien Lemal, Prof. Philippe Ghosez
Theoretical Materials Physics, Q-MAT, CESAM, Université de Liège, B-4000 Liège, Belgium

Dr. Zhenping Wu
State Key Laboratory of Information Photonics and Optical Communications and School of Science, Beijing University of Posts and Telecommunications, Beijing 100876, China

†Present address: Department of Applied Physics, Stanford University, Stanford, California 94305, USA

Field Effect

A "training" procedure was applied prior to gating experiments to avoid hysteresis behaviour in sheet resistance R_s as a function of back-gate voltage V_g [1, 2]: V_g was firstly swept from 0 V to the maximum positive voltage, +300 V. After the "training" step, as shown in Fig. S1, a reversible R_s vs. V_g behaviour can be observed. The curve was taken at 650 mK. We can see that for negative V_g (depletion regime), the change in R_s is not dramatic, as compared to the LAO-STO interface.

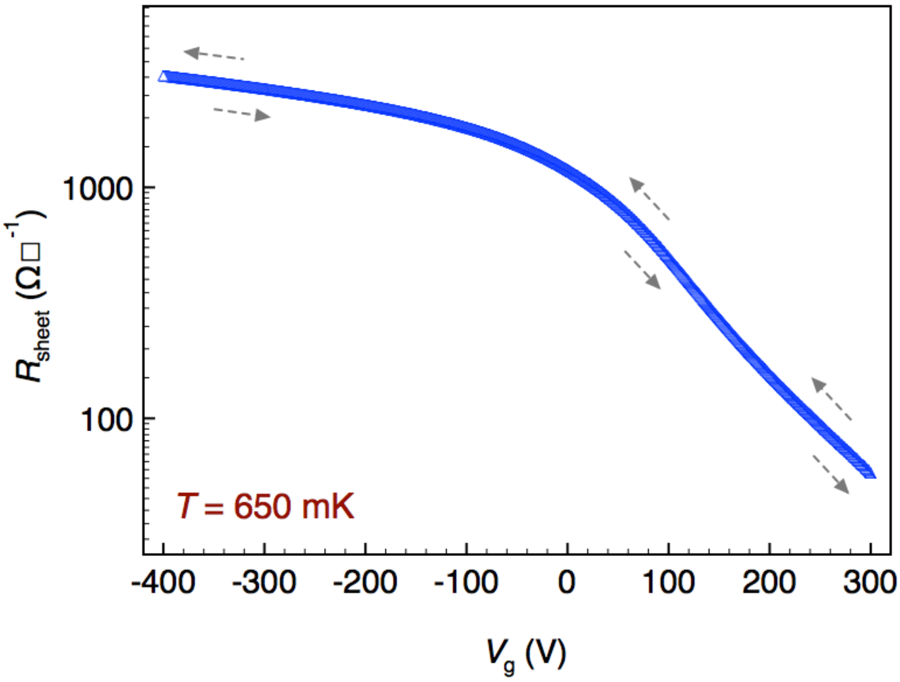

FIG. S1: Back-gate modulation of sheet resistance at the LASTO:0.5/STO interface. The curve is obtained after a "training" process by sweeping the gate voltage, V_g, from 0 V to +300 V at 650 mK. The dashed arrows indicate a reversible behaviour in V_g range of +300 V to −400 V.

P-S Model

Technical details

The general procedure of the self-consistent Poisson-Schrödinger model is briefly described below. The band mass and envelope function approximations are used. Parameters concern-

ing carriers from the n-th sub-band generated by the quantum confinement of the bulk band α are calculated. The indexed parameters used in the calculations are listed in Table S1.

TABLE S1: Definitions of the parameters used in the Poisson-Schrödinger calculation for different sub-band n generated by the quantum confinement of the bulk band α

	Definition
$m_i^{*,\alpha}$	Band mass for the bulk band α along i direction
ξ_n^α	Amplitude envelope function
ϵ_n^α	Solution of Eq. ES1 along $\hat{z}$
E_0^α	Energy bottom of the bulk band α
E_n^α	Energy levels of different sub-bands

A triangular potential well $V(z)$ that corresponds to a constant electric field in STO is taken as a starting point. The energy levels E_n^α for each band α in the well can be calculated using the two Eqs. ES1 and ES2:

$$-\frac{\hbar^2}{2m_z^{*,\alpha}}\frac{\partial^2\xi_n^\alpha(z)}{\partial z^2} + eV(z)\xi_n^\alpha(z) + E_0^\alpha\xi_n^\alpha(z) = \epsilon_n^\alpha\xi_n^\alpha(z) \tag{ES1}$$

$$E_n^\alpha(\mathbf{k}_\parallel) = \frac{\hbar^2k_x^2}{2m_x^{*,\alpha}} + \frac{\hbar^2k_y^2}{2m_y^{*,\alpha}} + \epsilon_n^\alpha \tag{ES2}$$

where Eq. ES1 is the Schrödinger equation. The solution of this equation ϵ_n^α can be used to calculate the energy levels. Furthermore, the Fermi energy E_F can be determined by Eq. ES3 setting the total sheet carrier density equal to $n_{2\mathrm{D}}$. This input value is 0.5 and 0.25 e$^-$/u.c. for LAO/STO and LASTO:0.5/STO interfaces, respectively.

$$n_{2\mathrm{D}} = \sum_{n,\alpha}\Theta(E_\mathrm{F}-\epsilon_n^\alpha)\frac{\sqrt{m_x^{*,\alpha}m_y^{*,\alpha}}}{\pi\hbar^2}(E_\mathrm{F}-\epsilon_n^\alpha) \tag{ES3}$$

with Θ the heaviside function. With this information the charge density $\rho_{3\mathrm{D}}$ is calculated using Eq. ES4:

$$\rho_{3\mathrm{D}}(z) = e\sum_{n,\alpha}\Theta(E_\mathrm{F}-\epsilon_n^\alpha)\frac{\sqrt{m_x^{*,\alpha}m_y^{*,\alpha}}}{\pi\hbar^2}(E_\mathrm{F}-\epsilon_n^\alpha)\xi_n^\alpha(z)^2 \tag{ES4}$$

and is used in the Poisson equation (ES5) to compute a new potential well that fits the charge distribution profile.

$$-\frac{\partial}{\partial z}(\epsilon_0\epsilon_r(E)\frac{\partial V(z)}{\partial z}) = \rho_{3D}(z) \tag{ES5}$$

This potential well $V(z)$ will then be re-injected into Eq. ES1. Multiple iterations are performed until a defined convergence criterion is satisfied (see Ref. [3] for details). As a result, the potential well is directly related to the sheet carrier density n_{2D}. We also note that, in Eq. ES5 enters the electric field dependence of the STO dielectric constant, $\epsilon_{STO}(E)$, which we discuss below.

Dielectric constant of STO

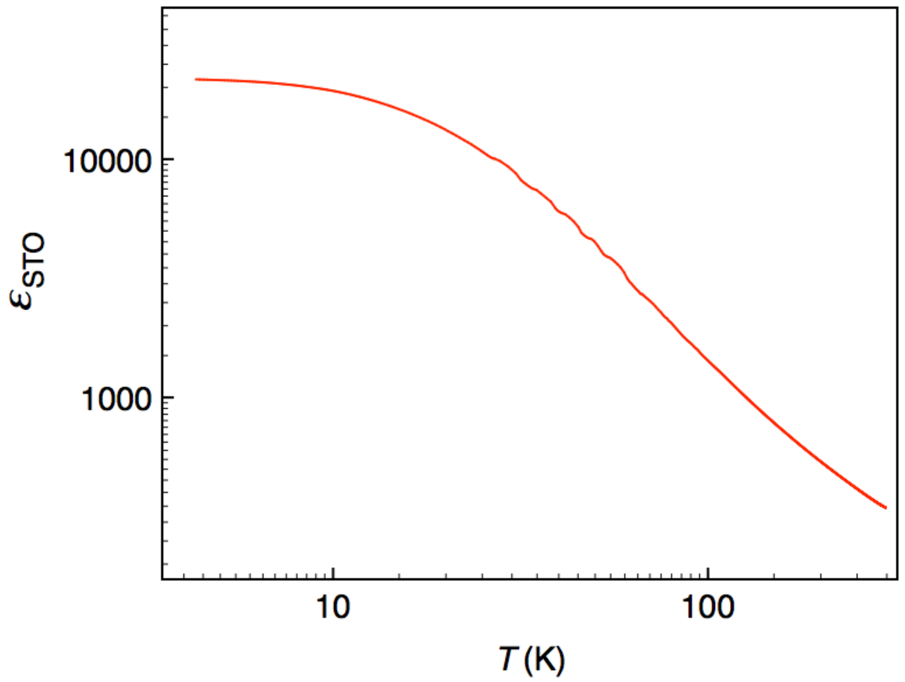

FIG. S2: Temperature evolution of the dielectric constant of STO, $\epsilon_{STO}(T)$, measured on STO single crystal using differential capacitance technique.

The temperature dependence of the dielectric constant of STO single crystal (0.5 mm thick), ϵ_{STO}, was measured and is shown in Fig. S2. Same as reported [4, 5], STO shows behaviour of an incipient ferroelectric as ϵ_{STO} increases dramatically upon cooling, especially

at low temperatures and plateaus at $\sim 10^4$ when approaching to 0 K. This remarkable enhancement of $\epsilon_{\rm STO}$ affects the confining potential at conducting oxide interfaces.

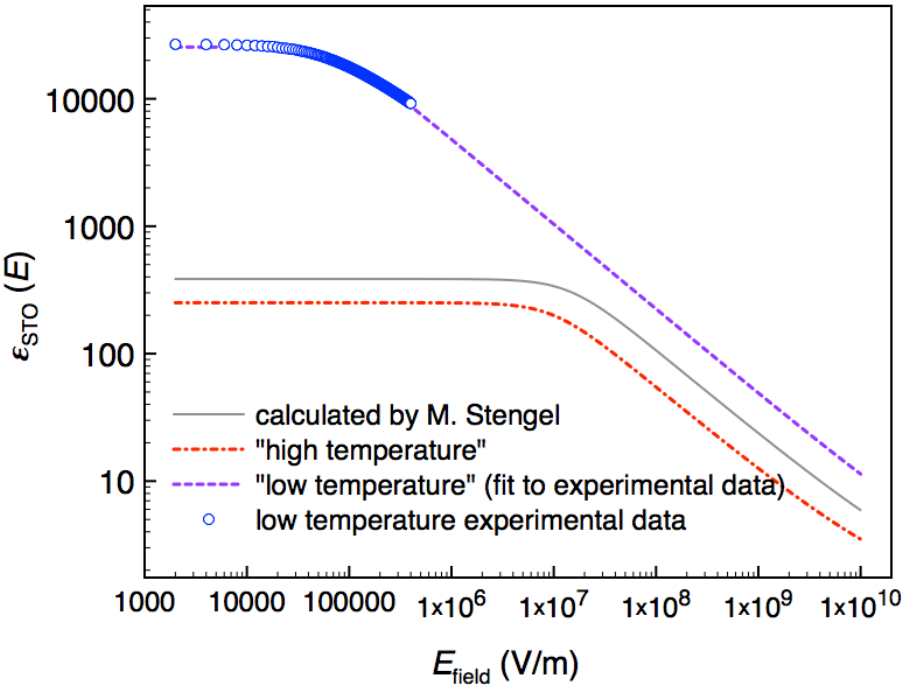

FIG. S3: Field dependence of the dielectric constant of STO, $\epsilon_{\rm STO}(E)$, used in P-S modelling. *Grey line*: calculation by M. Stengel [6]; *red dot-dashed line*: calculated data following [6] with a fixed low field $\epsilon_{\rm STO}$, which is obtained by DFT calculation (with equation used in Methods); *blue open circle*: experimental data measured at 4 K; *purple dash line*: fitting to low temperature experimental data using equation in Methods.

The electric field dependence of $\epsilon_{\rm STO}$ is shown in Fig. S3. As discussed in the main text, the static $\epsilon_{\rm STO}$ (~ 250) calculated by DFT corresponds to a room temperature low field value. The field dependence, $\epsilon_{\rm STO}(E)$, in this case, follows Eq. 3 shown in the main text and the calculation by Stengel [6]. $\epsilon_{\rm STO}(E)$ at low temperature has been experimentally measured (blue open circles in Fig. S3). A fit to the data gives $\epsilon_{\rm STO}(E)$ for the whole field range at low temperature, which also takes a similar form (Eq. 3 in the main text).

Confining potential

A large discrepancy in characteristic thickness and charge distribution for LASTO:0.5/STO and LAO/STO interfaces using low temperature $\epsilon_{\rm STO}(E)$ has been il-

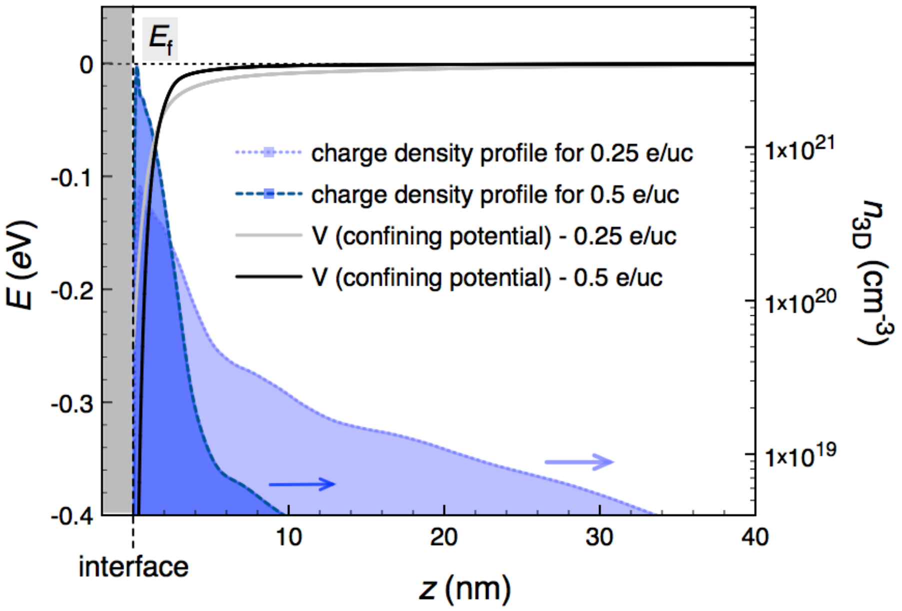

FIG. S4: Confining potential profiles for the low temperature case calculated by P-S model. *Right axis*: charge density profile for low temperature $\epsilon_{\mathrm{STO}}(E)$, same as in Fig. 3b; *left axis*: corresponding confining potential renormalised by Fermi energy.

lustrated in Fig. 3b in the main text. We plot here the corresponding confining potential profiles for both interfaces (0.25 e$^-$/u.c. and 0.5 e$^-$/u.c.). From the results we can see that the effect of charge de-confinement mostly become dramatic at the top of the confining well. This indicates that it is a small portion (compared to the total amount) of the transferred charges, which populates bands close to Fermi energy, that contributes to the large difference in 2DEL thickness.

Effective mass

To examine the effect of the effective mass m^* on the P-S modeling results, we performed the P-S calculation using a renormalized m^* (by a factor of 3.5, as compared to m^* obtained from DFT), which can originate from polaronic effect [7, 8]. The resulted charge distributions $n_{3\mathrm{D}}(z)$ using both "room-temperature" and "low-temperature" $\epsilon_{\mathrm{STO}}(E)$ are plotted in Fig. S5, as compared to Figure 3, which uses a m^* obtained from DFT calculations in the main text.

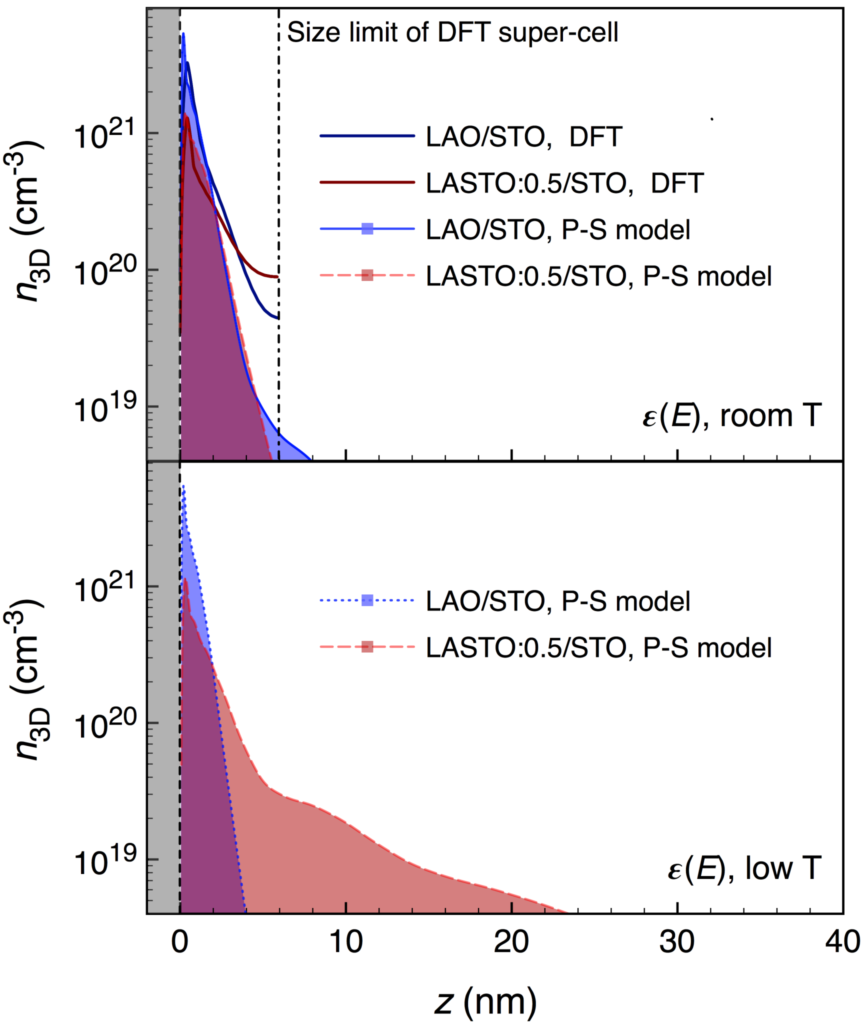

FIG. S5: Charge distributions in STO calculated for the two interfaces using renormalized m^* (by a factor of 3.5). The panels display the charge density ($n_{3\mathrm{D}}$) in logarithmic scale as a function of distance (z) from the interface for $n_{2\mathrm{D}}$= 0.5 e$^-$/u.c. (LAO/STO) and 0.25 e$^-$/u.c. (LASTO:0.5/STO). *Top panel*: Results from DFT calculations (solid lines) and P-S model (dotted/dash lines) for a room-temperature field-dependent STO dielectric constant $\epsilon_{\mathrm{STO}}(E)$. *Bottom panel*: Charge profile estimated with the P-S model (dotted/dash lines) using the low-temperature $\epsilon_{\mathrm{STO}}(E)$. Black dash line indicates the interface. Dot-dashed line corresponds to the size limit of the largest supercell used in DFT calculations.

DFT Calculations

In this work, we study off-stoichiometric superlattices of $(\mathrm{STO})_\mathrm{m}/(\mathrm{LAO})_2$ and $(\mathrm{STO})_\mathrm{m}/(\mathrm{LASTO{:}0.5})_2$, for full-compensated interfaces. In contrast to previous DFT stud-

ies, we do not explicitly remove charges from the interface; instead, the charge density is directly tuned through the composition of LASTO:x. The geometry of the studied systems is illustrated in Fig. S6, with $m = 12$ for clarity. We compute the structural and electronic properties of non-stoichiometric $(STO)_m/(LAO)_2$ and $(STO)_m/(LASTO{:}0.5)_2$ superlattices, with two n-type interfaces, and $m = 12,\ 22$ and 30. The non-stoichiometry is set by adding an additional TiO_2 and an additional LaO layers in their respective sub-lattice as shown in Fig. S6. The superlattice model is known to mimic fully compensated interfaces, using the additional AlO_2 planes to provide one single additional electron per 2D unit cell, which is shared by the two n-type interfaces, resulting in 0.5 e^-/a^2 manifested by the polar catastrophe scenario, i.e. $n_{2D} = 3.3 \times 10^{14}\ cm^{-2}$. Replacing the LAO layer with the LASTO:0.5 alloy only provide 0.5 additional electron in the cell, resulting in 0.25 e^-/a^2 as predicted by the polar catastrophe scenario.

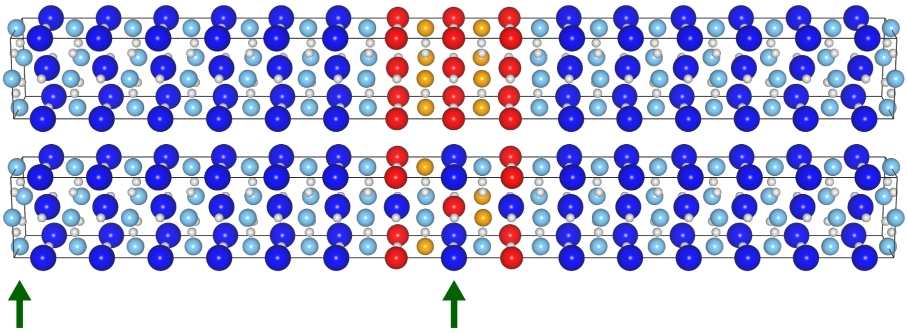

FIG. S6: Unit cell of the superlattice structure: $(STO)_{12}/(LAO)_2$ and $(STO)_{12}/(LASTO{:}0.5)_2$. Two additional planes of TiO_2 and LaO results in non-stoichiometry of the unit cell (green arrows).

Periodic boundary conditions are used. To mimic the substrate epitaxial strain, the in-plane cell parameter is fixed to the cubic bulk value of STO, $a_{STO} = 3.88$ Å, as determined in the framework of B1-WC. We use the tetragonal $P4/mmm$ space group for the superlattice with LAO interlayer, and $P\bar{4}m2$ space group for the superlattice with LASTO:0.5 interlayer, with an in-plane $\sqrt{2}a \times \sqrt{2}a$ supercell approach to accomodate the solid solution. In this manner, each supercell contains $2 \times (5[m + n + 1])$ atoms.

The present section aims to fully detail the results of our DFT calculations. As the superlattices are symmetric, the following results are only displayed for only one half of each superlattice.

Structural properties

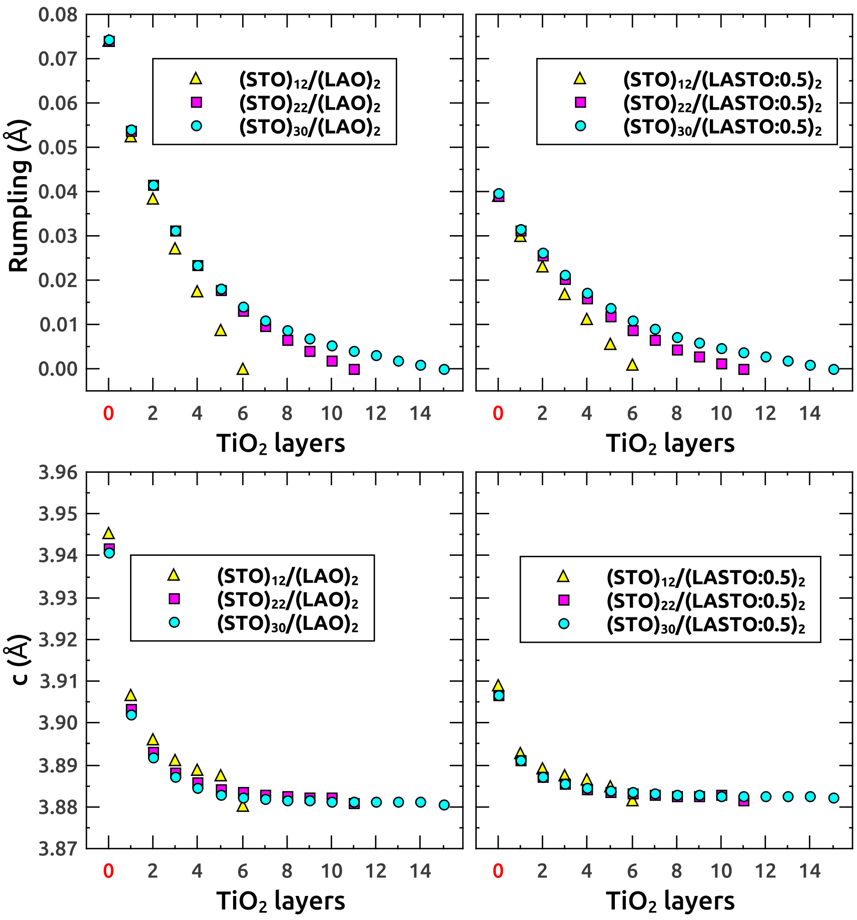

FIG. S7: Rumpling in TiO_2 plane in STO (0 is the TiO_2 interface), and out-of-plane lattice parameter c of STO.

The structural properties of the STO substrate are summarized in Figure S7, where the rumpling in TiO_2 planes (labeled from 0 to 15, with 0 being TiO_2 plane at the interface) and lattice parameter c are plotted, for the different superlattices. The rumpling is defined as the distance between cation and anion planes within a single atomic layer along [001] direction, while c is calculated as the distance between cation-anion median planes.

For all superlattices, the first observation is that c converges quickly to the lattice parameter of cubic bulk STO (determined by B1-WC). The second observation is that the rumpling also converges quickly toward 0 Å, retrieving $m\bar{3}m$ point group symmetry of bulk STO. The finite size of STO imposes a value of the rumpling in the last TiO_2 layer to be 0 Å, independently of the superlattice size, and the last value of c to be equal to a. The

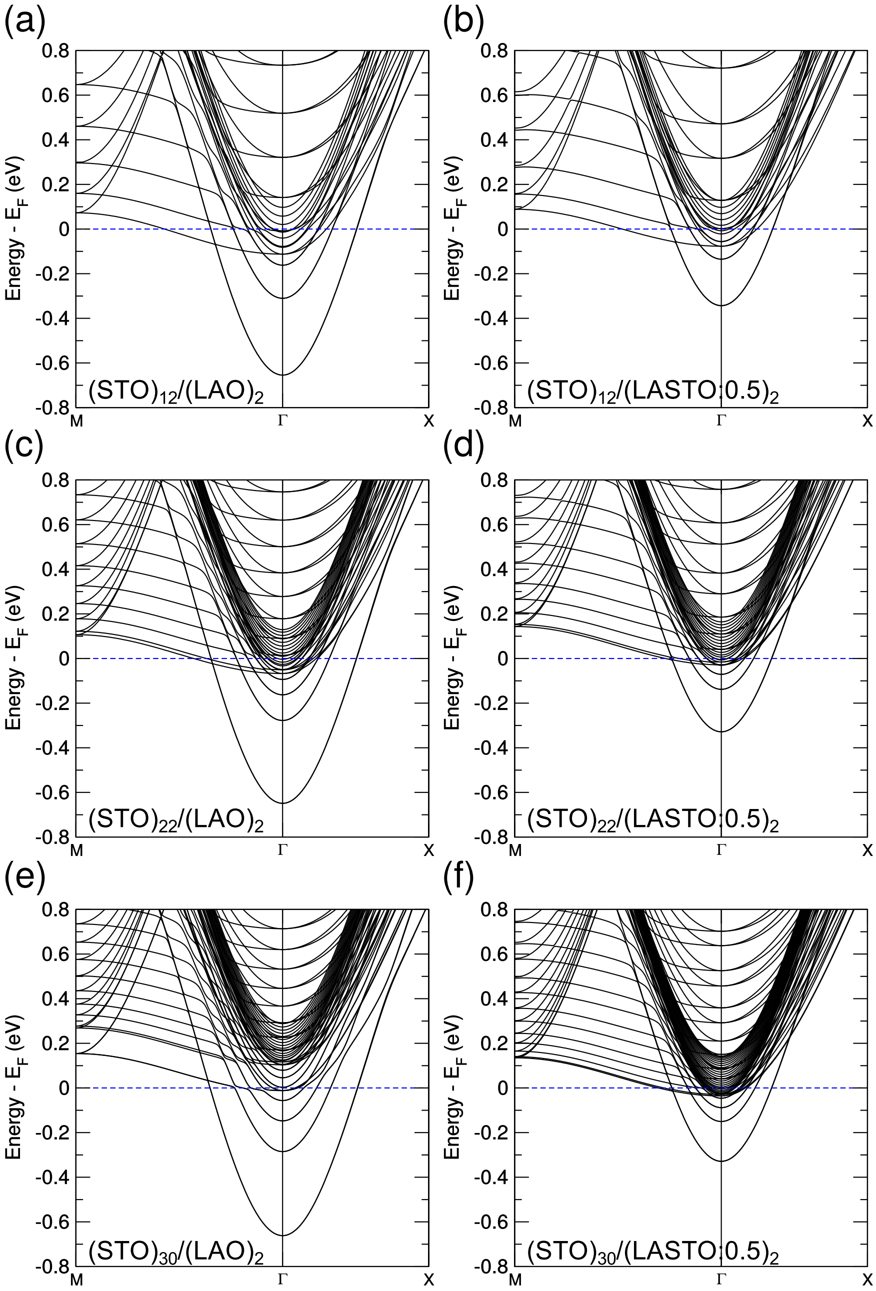

FIG. S8: Electronic band structure for $(STO)_m/(LAO)_2$ and $(STO)_m/(LASTO{:}0.5)_2$ superlattices with $m = 12$, 22 and 30. The Fermi level is shown by the horizontal dashed line.

out-of-plane strain applied to the individual cells of STO and the value of the rumpling depend on the amount of transferred charges.

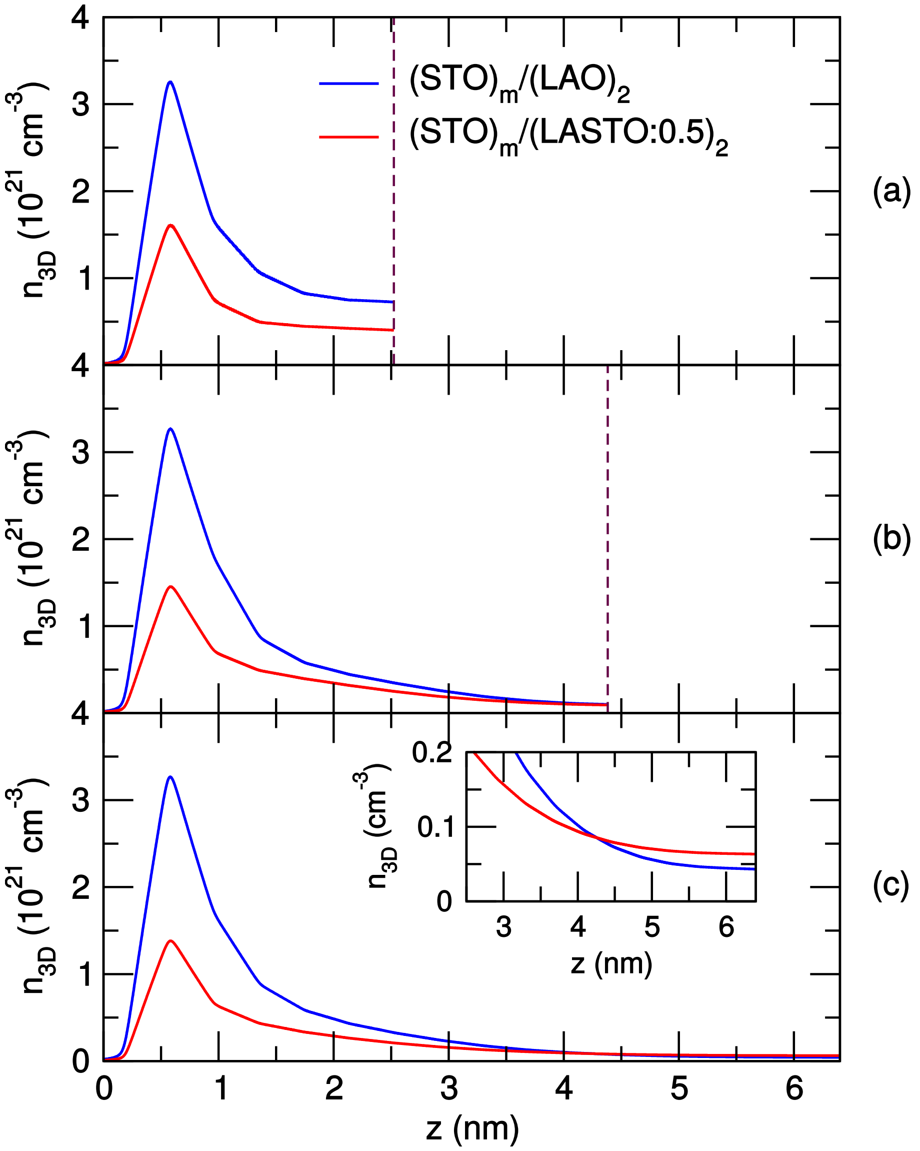

FIG. S9: Average macroscopic 3D electron density in $(STO)_m/(LAO)_2$ ($(STO)_m/(LASTO{:}0.5)_2$) superlattices for (a) $m = 12$, (b) $m = 22$ and (c) $m = 30$. The origin along z is taken as the middle of each superlattice. The vertical dashed lines indicate the middle of the STO sublattice.

Electronic properties

To get insights on the electronic properties, the electronic band structures for all superlattices are calculated, as illustrated in Fig. S8. The conduction band bottom is composed of $\mathrm{Ti} - d_{xz}$ and $\mathrm{Ti} - d_{xz}/d_{yz}$ states. The profile of the first available conduction states below Fermi level E_F, which is the first populated $\mathrm{Ti} - d_{xy}$ close to the interface, does not significantly evolve with m. Moving from LAO to LASTO:0.5 superlattices, the distance

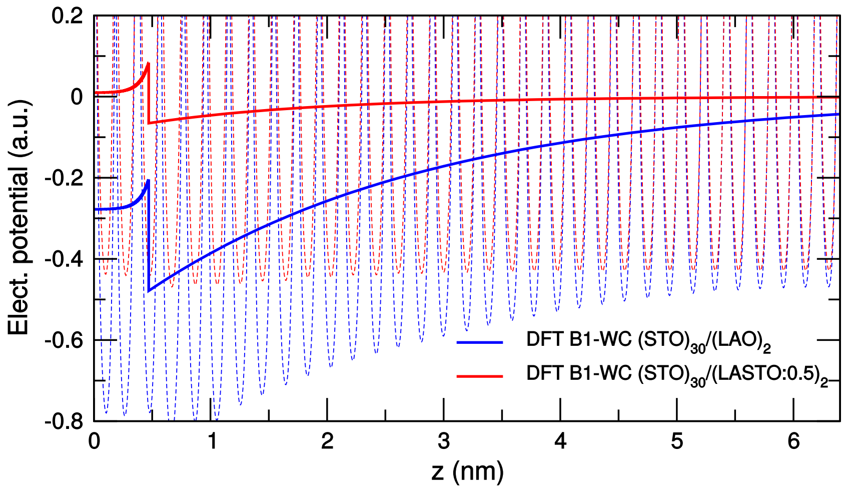

FIG. S10: Microscopic and averaged electrostatic potential in $(STO)_{30}/(LAO)_2$ and $(STO)_{30}/(LASTO{:}0.5)_2$.

between the conduction band minimum and E_F changes from 0.65 eV to 0.33 eV, which results in a change in electron density at the interface. For electrons occupying states far from the interface, only those that rest very close to E_F are relevant. Here m is found to be an important parameter: the thicker the STO slab in the superlattice is used, the denser the subband structure shows when close to E_F.

From the band structure, we calculate the effective masses, m^*, of Ti $-\,3d$ bands. In bulk STO, the DFT B1-WC effective masses for the light and heavy bands are $m_l = 0.4\ m_e$ and $m_h = 6.1\ m_e$, respectively. In the superlattices, the values calculated for the light and heavy carriers at the interface are $m_l = 0.4\ m_e$ and $m_h = 5.9\ m_e$, which are very close to their bulk values.

The average 3D electron density of the total transferred charges $n_{3D}(z)$ is plotted in Fig. S9 for $(STO)_m/(LAO)_2$ and $(STO)_m/(LASTO{:}0.5)_2$ ((a) $m = 12$, (b) $m = 22$ and (c) $m = 30$). $n_{3D}(z)$ is calculated by averaging the electron density in the ab plane, as moving along z. Several observations can be made from these figures: (i) firstly, the density at the TiO_2 interface does not evolve with increasing m, suggesting that the superlattice model properly captures the properties at the interfaces, as calculations performed in previous work. This is in consistency with the profile of the first occupied conduction band in STO, which does not change with m; (ii) secondly, far from the interfacial TiO_2 plane, n_{3D} converges very slowly with m. This is identified as size effect in the STO sublattice: the transferred

TABLE S2: Mulliken decomposition of the transferred charges in the STO $Ti-3d$ orbitals for superlattices with $m = 30$. In this picture, the remaining transferred charges, which are not found in $Ti-3d$ orbitals, are located in the $O-2p$ states within TiO_2 planes.

	**LAO**		**LASTO:0.5**	
**Ti**	$\mathbf{d_{xy}}$	$\mathbf{d_{xz}+d_{yz}}$	$\mathbf{d_{xy}}$	$\mathbf{d_{xz}+d_{yz}}$
15	0.001	0.002	0.001	0.002
14	0.001	0.002	0.001	0.002
13	0.001	0.002	0.001	0.002
12	0.001	0.002	0.001	0.002
11	0.001	0.002	0.001	0.002
10	0.001	0.002	0.001	0.002
9	0.001	0.004	0.001	0.004
8	0.002	0.004	0.001	0.004
7	0.002	0.006	0.002	0.004
6	0.004	0.008	0.002	0.006
5	0.006	0.010	0.004	0.006
4	0.010	0.012	0.006	0.008
3	0.018	0.012	0.012	0.007
2	0.038	0.010	0.017	0.006
1	0.085	0.006	0.031	0.004
0	0.177	0.002	0.073	0.002
Σ	0.349	0.086	0.154	0.063
Σ $Ti-3d$	0.435		0.217	
% $Ti-3d$	71%	17%	63%	25%
Σ Rest	0.060		0.032	
% Rest	12%		12%	

electron charge cannot relax further beyond the boundary that is set by the size of the

supercell (the boundary is indicated as the vertical dashed line); iii) finally, substituting LAO with LASTO:0.5 decreases the n_{3D} at the interface, but electrons extend further, as illustrated in Fig. S9c. The crossing of the two curves occurs at 4.25 nm from the middle of the superlattice.

Despite the different sizes of STO in the superlattices, the n_{3D} never properly reaches 0. For $m = 30$, the minimum density (in $(STO)_{30}/(LAO)_2$) in the STO sublattice is 4.3×10^{19} cm^{-3}. For $m = 12$ or 22, the de-confinement of the electrons far from the interface cannot be resolved. Hence, the results for $m = 30$ suggest that, if a STO with larger size is implemented in the calculation, we should observe a stronger de-confinement of the electrons from the interface for LASTO:0.5/STO system.

The confining potential in STO depends on the density of the total transferred charges. This is illustrated in Fig. S10, in which the electrostatic potential is plotted for both $(STO)_{30}/(LAO)_2$ and $(STO)_{30}/(LASTO{:}0.5)_2$ interfaces. The electric field in STO decreases moving from charge density of 0.5 to 0.25 e/a^2. The confinement of the transferred electrons is therefore also reduced.

To have a complete grasp on the occupation of the different $Ti - 3d$ orbitals, we list in Table S2 the orbital-resolved Mulliken population of the transferred electrons for both $(STO)_{30}/(LAO)_2$ and $(STO)_{30}/(LASTO{:}0.5)_2$ interfaces. Within this scheme, 88% of the total electrons occupy $Ti - 3d$ orbitals. The rest is attributed to $O - 2p$ states. The different populations reflect the occupation of the associated band in Fig. S8. The d_{xy} population decreases very quickly from the first two interfacial TiO_2 planes into STO. The tail of charge distribution is mainly dominated by electrons with $d_{xz} + d_{yz}$ character. When the density of the total transferred electrons is reduced, the relative proportion of the $d_{xz} + d_{yz}$ slightly increases.

[1] Caviglia, A. D. *et al.* Electric field control of the $LaAlO_3/SrTiO_3$ interface ground state. *Nature* **456**, 624–627 (2008).

[2] Liu, W. *et al.* Magneto-transport study of top-and back-gated $LaAlO_3/SrTiO_3$ heterostructures. *APL materials* **3**, 062805 (2015).

[3] Fête, A. *Magnetotransport experiments at the $LaAlO_3/SrTiO_3$ interface*. Ph.D. thesis, Univer-

sity of Geneva (2014).

[4] Sakudo, T. & Unoki, H. Dielectric properties of $SrTiO_3$ at low temperatures. *Physical Review Letters* **26**, 851–853 (1971).

[5] Müller, K. A. & Burkard, H. $SrTiO_3$: an intrinsic quantum paraelectric below 4 K. *Physical Review B* **19**, 3593–3602 (1979).

[6] Stengel, M. First-principles modeling of electrostatically doped perovskite systems. *Physical Review Letters* **106**, 136803 (2011).

[7] Cancellieri, C. *et al.* Polaronic metal state at the $LaAlO_3/SrTiO_3$ interface. *Nature communications* **7**, 10386 (2016).

[8] Dubroka, A. *et al.* Dynamical response and confinement of the electrons at the $LaAlO_3/SrTiO_3$ interface. *Physical Review Letters* **104**, 156807 (2010).
